# Single-Shot Detection of Neurotransmitters in Whole-Blood Samples by Means of the Heat-Transfer Method in Combination with Synthetic Receptors

**DOI:** 10.3390/s17122701

**Published:** 2017-11-23

**Authors:** Thijs Vandenryt, Bart van Grinsven, Kasper Eersels, Peter Cornelis, Safira Kholwadia, Thomas J. Cleij, Ronald Thoelen, Ward De Ceuninck, Marloes Peeters, Patrick Wagner

**Affiliations:** 1 Institute for Materials Research, Hasselt University, Wetenschapspark 1, B-3590 Diepenbeek, Belgium; thijs.vandenryt@uhasselt.be (T.V.); bart.vangrinsven@maastrichtuniversity.nl (B.v.G.); kasper.eersels@maastrichtuniversity.nl (K.E.); peter.cornelis@kuleuven.be (P.C.); ward.deceuninck@uhasselt.be (W.D.C.); m.peeters@mmu.ac.uk (M.P.); patrick.wagner@fys.kuleuven.be (P.W.); 2 Maastricht Science Programme, Maastricht University, P.O. Box 616, 6200 MD Maastricht, The Netherlands; thomas.cleij@maastrichtuniversity.nl; 3 Department of Physics and Astronomy, Soft-Matter Physics and Biophysics Section, KULeuven, Celestijnenlaan 200 D, B-3001 Leuven, Belgium; 4 Division of Chemistry and Environmental Science, School of Science and the Environment, Faculty of Science and Engineering, Manchester Metropolitan University, Chester Street, Manchester M1 5GD, UK; safira.kholwadia@gmail.com; 5 IMOMEC Division, IMEC vzw, Wetenschapspark 1, B3590 Diepenbeek, Belgium

**Keywords:** heat-transfer method, biomimetic sensing, molecularly imprinted polymers, neurotransmitters, point-of-care diagnostics

## Abstract

Serotonin is an important neurotransmitter that plays a major role in the pathogenesis of a variety of conditions, including psychiatric disorders. The detection of serotonin typically relies on high-performance liquid chromatography (HPLC), an expensive technique that requires sophisticated equipment and trained personnel, and is not suitable for point-of-care applications. In this contribution, we introduce a novel sensor platform that can measure spiked neurotransmitter concentrations in whole blood samples in a fast and low-cost manner by combining synthetic receptors with a thermal readout technique—the heat-transfer method. In addition, the design of a miniaturized version of the sensing platform is presented that aims to bridge the gap between measurements in a laboratory setting and point-of-care measurements. This fully automated and integrated, user-friendly design features a capillary pumping unit that is compatible with point-of-care sampling techniques such as a blood lancet device (sample volume—between 50 µL and 300 µL). Sample pre-treatment is limited to the addition of an anti-coagulant. With this fully integrated setup, it is possible to successfully discriminate serotonin from a competitor neurotransmitter (histamine) in whole blood samples. This is the first demonstration of a point-of-care ready device based on synthetic receptors for the screening of neurotransmitters in complex matrices, illustrating the sensor’s potential application in clinical research and diagnosis of e.g., early stage depression.

## 1. Introduction

Mood disorders, in particular depression with a lifetime prevalence of 15–20%, are the most common form of psychiatric disorders in Europe [1,2]. In 2010, nearly 7% of the European population had been diagnosed with major depression, and 1–2% with bipolar depression [3]. The financial burden on the healthcare system mounted up to 113.4 billion Euroes in that year, making it the most important single contributor to the total EU disease-related healthcare cost [4]. It is known that for many psychiatric disorders, neurotransmitters and their receptors play a major role in the pathogenesis [5,6]. In particular, the balance between the neurotransmitters dopamine and serotonin is of important significance when developing novel treatments or medications [7,8]. Although serotonin cannot be transported across the blood-brain barrier, anomalous whole blood serotonin levels are correlated with clinical depression [9,10,11], and timely intervention in a personalized medicine-based setting can significantly reduce the associated medical and socio-economics burdens [12,13]. Furthermore, serotonin is involved in steering numerous behavioral and physiological functions and abnormalities in serotonin levels are also found in patients with e.g., hypertension [14] and gastrointestinal disorders such as irritable bowel syndrome (IBS) [15]. 

The most common technique for detecting serotonin in patient samples is based on high-performance liquid chromatography (HPLC) as, in contrast to other techniques, it can selectively discriminate between different neurotransmitters [16,17,18]. However, HPLC suffers from financial drawbacks, as it requires sophisticated equipment that needs to be used in a lab environment. In addition, sample handling, device operation, and data analysis are challenging and require trained personnel, rendering HPLC unsuitable for straightforward routine tests. Measurements have to be carried out under stringently controlled conditions as serotonin belongs to a class of molecules that are very sensitive to light, oxygen, and changes in pH [19,20]. 

In previous research, a thermal sensor platform was developed that could selectively measure serotonin in buffer solutions in the physiologically relevant regime [21,22]. This concept was based on combining synthetic receptors, molecularly imprinted polymers (MIPs), with a novel read-out strategy, the heat-transfer method (HTM). MIPs are able to detect chemical substances in complex matrices and have the advantages of facile and cheap synthesis, high chemical and thermal stability, re-usability, and an almost unlimited shelf life [23,24,25]. When template molecules bind to the MIP receptor layer, the thermal resistance increases and detection of serotonin in buffer solutions in the low nanomolar regime was demonstrated [21]. The thermal readout platform could be combined with various other receptors leading to other biosensing applications, such as DNA mutation analysis, phase changes in lipids, and detection of cells, but biological samples remained to be evaluated [26,27,28]. 

In this contribution, a four chamber HTM sensor platform was used for the detection of serotonin in spiked whole blood samples. The concept of quantitative serotonin detection in whole blood was established by exposing the sensor to whole blood samples spiked with increasing concentrations of serotonin. The results of these experiments show that the sensor is able to detect differences in serotonin concentrations in the 100 nM range which fit nicely into the physiological regime of 100 nM to 10 µM with whole blood serotonin levels in patients and non-patients differing on average by several hundreds of nanomolar [11]. Selectivity and specificity were demonstrated by means of a cross-selectivity measurement using histamine as an analogue and a reference measurement on a non-imprinted polymer respectively. 

In order to bring the technology one step closer to point-of-care diagnostic applications, a miniaturized single-shot device was designed and tested. This device features an integrated capillary pumping system that allows for autonomous, passive administration of blood samples, thereby overcoming the need for an elaborate active pumping system. This facilitates miniaturization. Furthermore, the single-shot device is user-friendly because sample pre-treatment is limited to merely the addition of an anti-coagulant, and the small sampling volume renders the sensor compatible with standard blood sampling techniques such as a blood lancet device. Quantitative detection and selectivity are also demonstrated in this single-shot device thereby demonstrating the first hands-on, disposable device based on synthetic receptors that can be used for the detection of neurotransmitters in complex matrices. In this way, we illustrate the potential application of the device in clinical research in general and early stage depression diagnosis in particular.

## 2. Materials and Methods

### 2.1. Design of the Four Chamber-HTM Platform

**Figure 1:**
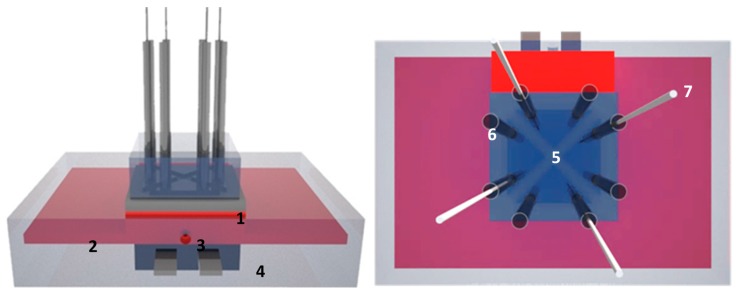
Schematic design of the four-chamber heat-transfer method (HTM) setup: (**Left panel**) overview: the sensor chip (**1**) was placed on a copper lid (**2**), which was used as a heat sink. The central cavity in the copper lid is intended to install a thermocouple (**3**). The assembly is heated by power resistor (**4**). (**Right panel**) top view: the sensor was divided into four triangular shaped identical sections of 10 mm^2^ (**5**) with a height of 1 mm by means of a polydimethylsiloxane (PDMS) flow cell. Each flow cell connects to two Teflon tubes serving as fluid in- and outlet (**6**) and thermocouple holders (**7**), respectively. An alternative 2D representation of the setup can be found in [22].

To demonstrate the proof-of-principle of measuring neurotransmitters in whole blood samples, a sensor setup was developed that measured the heat-transfer resistance at the solid-liquid interface (Figure 1). A similar design has been described before for the detection of histamine with MIPs grafted onto graphene oxide [29] and consists of a polydimethylsiloxane (PDMS) flow cell with four identical chambers of 1 µL each, allowing simultaneous measurements on four spots of the sample. 

Within the proof-of-principle measurements, whole blood samples were exchanged my means of syringes connected to the tubing system. The device was equipped with five miniaturized thermocouples (type K, diameter 500 μm, TC Direct, The Netherlands) monitoring the temperature *T*_1_ of the copper backside contact and the liquid temperatures *T*_2,3,4,5_ in each PDMS segment at a height of 0.5 mm above the chip surface. The heat flow was generated by a power resistor (22 Ohm, MPH 20, Farnell, Bierset, Belgium) applied onto the copper block and soldered in place to improve the thermal contact. To regulate *T*_1_, the thermocouple signal was led to a data acquisition unit (Picolog TC08, Picotech, Cambridgeshire, UK) and from there processed into a PID controller (parameters: P = 1, D = 8, I = 0). The calculated output voltage was sent via a second controller (NI USB 9263, National Instruments, Austin, TX, USA) to a power operational amplifier (LM675, Farnell, Bierset, Belgium) and fed into the power resistor. Sampling of the *T*_1_ and *T*_2,3,4,5_ values was done at a rate of one measurement per second. The sensing concept was based on a temperature gradient, which was generated by applying a heating power (*P*) at the copper backside. The thermal resistance (*R_th_*) was obtained from the temperature difference between the copper backside (*T*_1_) and the liquid (*T_n_* with *n* = 2, 3, 4, 5) with *T_n_* relating to the four different measurement spots in the liquid. The difference in temperature between the heat sink and each of the four fluid compartments (*T*_1_ − *T_n_*) was divided by the power (*P*) needed to heat up the system to obtain the heat transfer resistance in °C/W (Equation (1)). In previous research, it has been shown that the thermal resistance is highly sensitive to the properties at the solid-liquid interface [30].

(1) $$R_{th}=\frac{T_{1}-T_{n}}{P}\;\mathrm{with}\;n=2,3,4,5$$

### 2.2. Design of a Single-Shot Device

**Figure 2:**
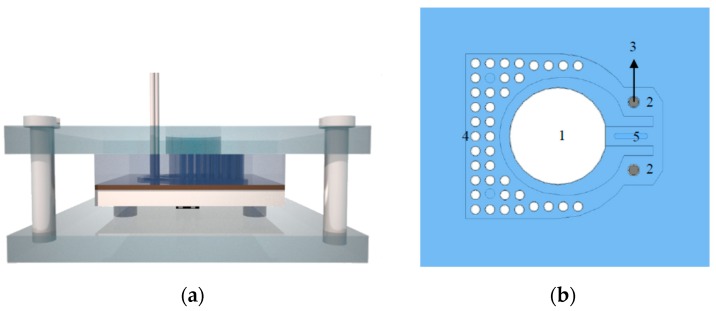

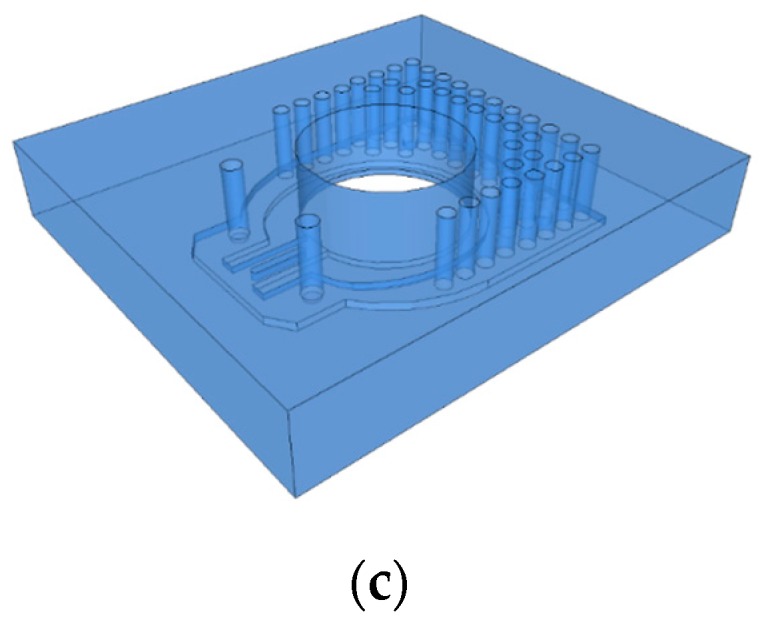
Single-shot measurement setup (**a**) Front view: consists of: a heater module, a selective deposited molecularly imprinted polymer (MIP) layer on an aluminum substrate, a PDMS flow cell and 3 temperature sensors which is here shown as a cross section of the finished flow cell. (**b**) The flow cell occupies a total area of 30 × 30 mm^2^ and contains one centralized inlet (**1**), two parallel sensing areas of 9 mm^2^ each (**2**) with integrated thermocouples (**3**) and a pumping area (**4**). The volumetric flow rate of the sample fluid can be controlled by changing the width of the channel in position (**5**). (**c**) The height of the flow cell is 0.6 mm, and the vertical pump is 8 mm high. Whole blood samples can be added to the inlet. The measurements can be carried out as single-shot, without a stabilization step in buffer solution.

After demonstrating a proof-of-principle for serotonin detection in whole blood using the four-chamber HTM device, the flow cell needed to be optimized toward point-of-care applications. Therefore, a user-friendly design was developed that allows measuring neurotransmitters in a single-shot manner with a fully integrated set up. The flow cell was constructed from PDMS cast from a Teflon master mold (30 × 30 mm^2^), with a height of 0.6 mm. The design featured a vertical capillary pump array and two measurement areas (3 × 3 mm^2^), making it possible to measure the MIP and reference channel simultaneously. The 38 vertical capillary pumps, with a diameter of 0.5 mm, allowed a smooth continuous flow over the measurement areas [31] and could hold a large sample volume combined with a small footprint (Figure 2). The contact angle between untreated PDMS and aqueous solutions is high (>100°), therefore, no spontaneous capillary transport can occur. Common methods to make the polymer more hydrophilic are surface modifications such as plasma and corona treatment. These methods activate the surface by creating dangling bonds which can interact with the water molecules. A major drawback is that this activation only lasts for a couple of hours when stored in air and is therefore not suitable for this application [32]. A surface modification method with long-term stability was demonstrated by Yao and Fang, who used a block copolymer of PDMS in which ethylene oxide chains are embedded; Poly(dimethylsiloxane-*b*-ethylene oxide) (PDMS-*b*-PEO) [33]. A similar approach was used in this paper by mixing PDMS-*b*-PEO with uncured base polymer in a 1.3 wt % ratio, which lowers the contact angle to 45°, allowing spontaneous capillary transport. This PDMS flow cell was held together by a transparent Perspex casing that clamps the set up on top of the aluminum sensor substrate, which in turn was pressed onto the heat-spreader and heater. The modified PDMS continued to be self-sealing, so no permanent bonding of layers was required. One main inlet was provided, able to deposit up to 300 µL of the sample liquid. Two type-K thermocouples were permanently installed, 0.2 mm above the measurement area instead of 0.5 mm above the surface area as in the four-chamber set up, making it possible to determine the properties at the solid-liquid interface more precisely. Furthermore, the principle and electronic readout equipment was identical to the four-chamber design. The temperature gradient between the liquid and the heat provider is divided by the applied power, to calculate the *R_th_* for the active MIP-functionalized channel and the reference channel containing non-imprinted polymer (NIP) particles.

### 2.3. Receptor Layer

The polymerization of the MIP for serotonin has been described in [34]. In brief, a mixture of 2.84 mmol methacrylic acid (MAA), 8.5 mmol acrylamide (AM), 22.72 mmol ethylene glycol dimethacrylate (EGDMA), and 0.61 mmol azobisisobutyronitrile (AIBN) was dissolved into 7 mL of porogen dimethylsulfoxide (DMSO for HPLC, ≥99.7%) together with the template molecule serotonin hydrochloride (5.67 mmol). All chemicals were ordered at Sigma Aldrich (Gillingham, UK). This solution was purged with N_2_ and thermal polymerization was achieved by placing the mixture in an oven at 65 °C for 12 h. After polymerization, the bulk polymer was ground to obtain the microparticles. Serotonin was removed from the MIP powders by Soxhlet extraction with methanol (48 h), a mixture of acetic acid / acetonitrile (1/1) (48 h) and again methanol (12 h). All solvents were obtained from Sigma Aldrich (Gillingham, UK) and had a purity of at least 99%. The resulting powders were then dried in vacuum for 12 h. The reference NIP was synthesized accordingly but without the presence of the target molecule and could therefore serve as a reference. Aluminum chips were cleaned in acetone, isopropanol, and MilliQ before they were functionalized with an adhesive polyvinyl chloride layer by spin coating at 5000 rpm at an acceleration of 1650 rpms/s to yield a layer of 400 nm. The adhesive layer was heated to 120 °C, well above its glass transition temperature, for 120 s in order to enable stamping of MIP and NIP particles. Different measuring chambers were stamped by means of a home-made stamping device; residual unbound particles were removed by rinsing with Milli Q and blow drying using compressed N_2_. For more details on the procedure, we refer to [34].

### 2.4. Proof-of-Principle Experiments in Whole Blood Samples

An aluminum chip (10 × 10 mm^2^) was coupled to the four chamber-flow cell dividing the surface into four equal triangular parts. The four channels were coated with serotonin MIPs. Whole blood samples were obtained from a healthy volunteer and transferred to a Vacuette blood collection tube (Greiner Bio-One B.V., Alphen a/d Rijn) containing sodium citrate as an anti-coagulant. The samples were divided into different aliquots and spiked with increasing concentrations (100, 200, 300, 500, 750 and 1000 nM) of the target, serotonin, and an analogue histamine. Measurements were initiated after the signal was left to stabilize for ±20 min by adding 80 µL of the spiked blood samples sequentially to each fluid compartment using the Teflon tubing (20 µL/chamber). Between each addition step the signal was allowed to stabilize for 10–20 min until a constant value was reached. The heat-transfer resistance was derived in each step using Equation (1). Reference measurements were done using a NIP reference and a dose-response curve for each of the experiments. 

### 2.5. Single-Shot Serotonin Measurement in Whole Blood Samples

The fully integrated single-shot device consisted out of two identical sections; one was used as an active MIP-functionalized channel, while the other served as a reference (Figure 2). Blood samples were obtained in a manner similar as described in the previous section, and spiked with serotonin and histamine (1000 nM) to assess selectivity. To apply the sample, two droplets of blood (~80 µL) were added to the central cavity of the set up. The droplets reached the receptor layer in less than 15 s due to the capillary force effect, after which serotonin rebinding to the MIP receptor layer was registered by the setup. In addition, quantitative determination of serotonin was assessed in a similar manner exposing the sensor to increasing concentrations of serotonin (100, 200, 300, 500, 750, and 1000 nM).

### 2.6. Optical Characterization

The morphology of the synthesized MIP particles and their surface coverage on the measurement chips were analyzed using a FEI Quanta 200 FEG, Scanning Electron Microscope (SEM).

## 3. Results and Discussion

### 3.1. Proof-of-Principle: Thermal MIP-Based Detection of Serotonin in Whole Blood

**Figure 3:**
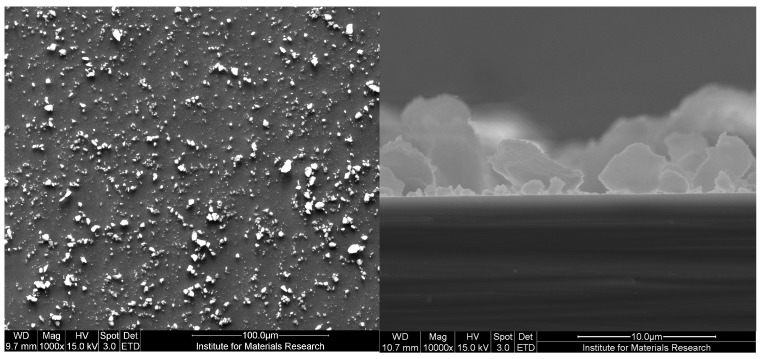
Scanning electron microscope (SEM) analysis of a MIP-coated aluminum chip (**left**) and a cross-section analysis of the same sample (**right**).

The SEM analysis shown in Figure 3 illustrates the typical heterogeneous morphology of MIPs made by bulk imprinting polymerization and indicates that the size selection by the sieving process was successful. The surface coverage on the measurement chips is evenly distributed and the cross-section analysis illustrates that the particles are indeed embedded into the adhesive layer.

### 3.2. Proof-of-Principle: Thermal MIP-Based Detection of Serotonin in Whole Blood

**Figure 4:**
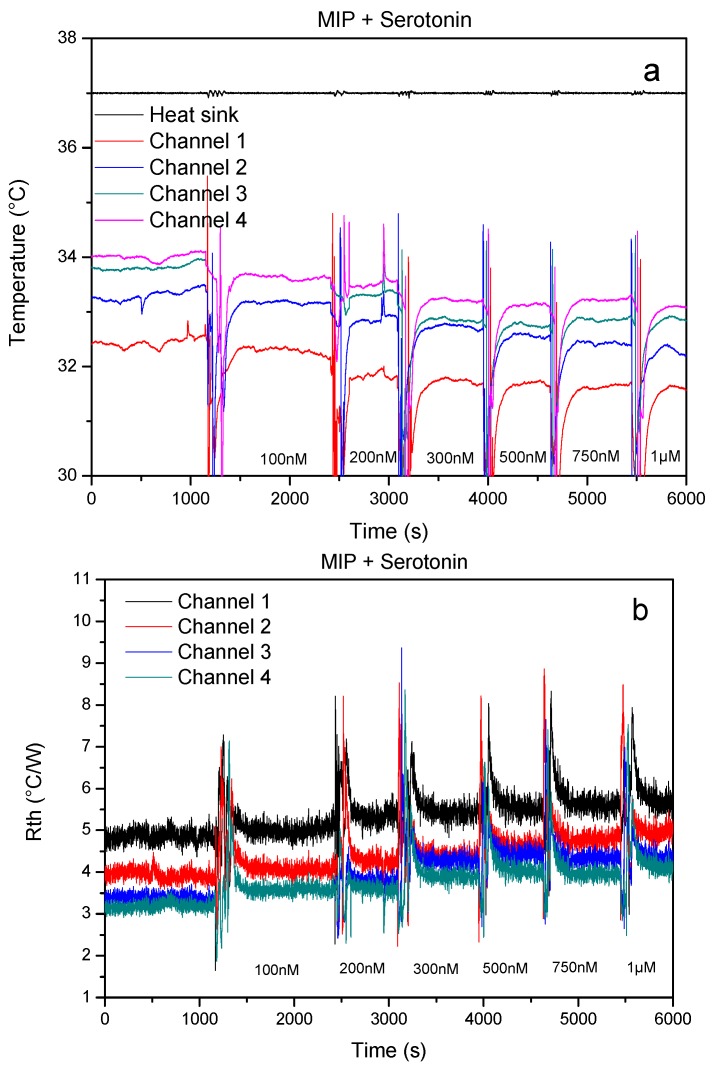
Results obtained in a proof-of-principle experiment using the four-chamber HTM device (**a**) shows the temperatures *T*_1,2,3,4,5_ as function of time, (**b**) shows the corresponding time-dependent heat-transfer resistance. A concentration dependent effect on both the temperature and thermal resistance signal can clearly be observed.

The measurements were conducted as described in the Section 2.4 and the resulting time-dependent thermal analysis is summarized in Figure 4. After stabilization of the signal, blood samples spiked with increasing concentrations were added to the measuring chambers, leading to a concentration-dependent decrease in the non-regulated temperatures (Figure 4a). The resulting thermal resistance analysis indicates that *R_th_* increases in function of the concentration of target molecule present in the measuring chamber (Figure 4b). These results are in line with previous findings, binding of the target will partially block the heat transport through the solid-liquid interface, which causes the temperature inside the flow cell to drop.

**Figure 5:**
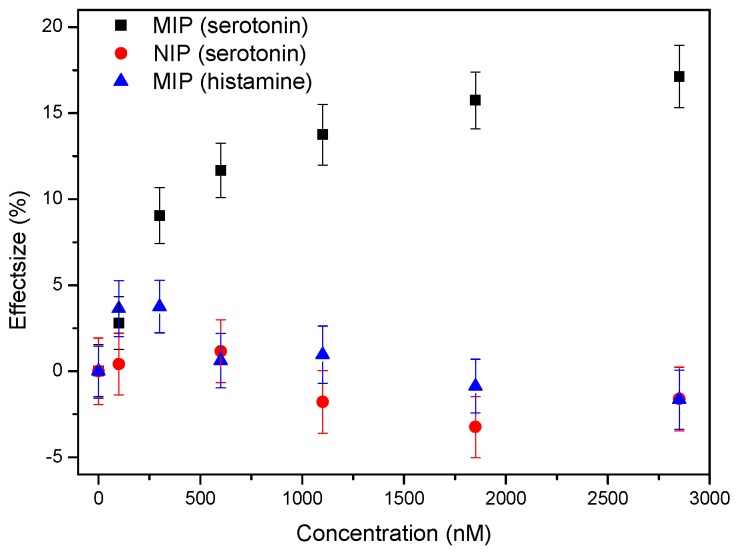
Dose-response curve obtained from the experiment described in Figure 4 (**black curve**), reference experiments on NIP-coated chips (**red curve**), and selectivity (**blue curve**).

To assess specificity of the synthetic receptor layer, NIP-coated chips were also exposed to increasing concentrations of serotonin in a similar experiment. Selectivity was analyzed by studying the response of a MIP-coated chip to an increasing concentration of an analogue neurotransmitter, histamine. The results of these experiments were summarized and compared to the specific response derived from Figure 4 in Figure 5. The dose-response curve indicates that there is not specific effect observed in both of the reference measurements, while significant increases in the thermal resistance effect already become apparent at concentrations of ±400 nM for the MIP-coated samples after which a stepwise increase can be observed spanning the higher nanomolar and low micromolar range. These results indicate that the sensor could potentially be used in the future to study serotonin-related diseases by differentiating between patients and non-patients were the whole blood serotonin levels often vary by several tens to several hundreds of nanomolar [11]. Improving the surface coverage and imprinting procedure by using more controllable polymerization strategies [29] could further improve the detection limit, resolution, and dynamic range of the current platform. 

### 3.3. Single-Shot Detection of Serotonin in Whole Blood Samples

**Figure 6:**
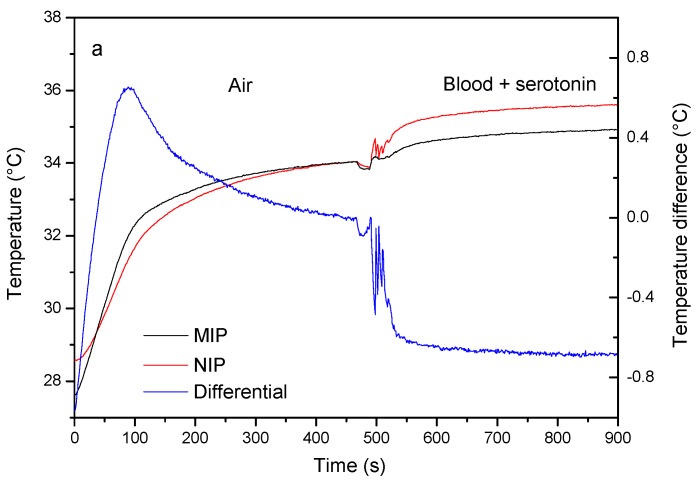

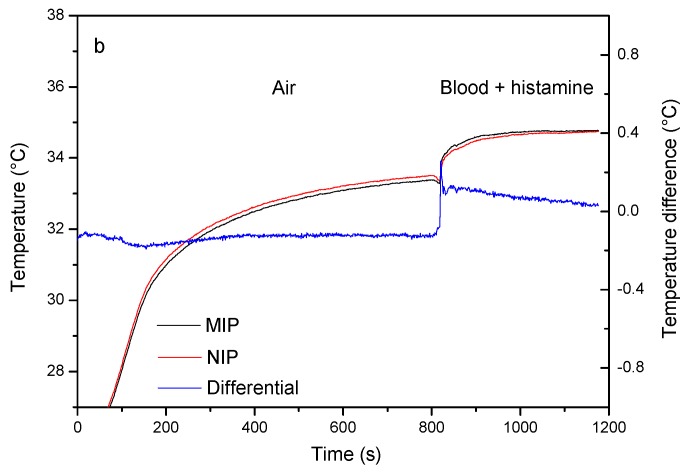
Results obtained using the single-shot device (**a**) 300 µL blood, spiked to a concentration of 1 µM of serotonin is added to the central cavity. An increase in temperature can be observed in both the NIP (**red curve**) and MIP (**black curve**) channel due to the medium change. The increase in the MIP is less pronounced as serotonin binds to the MIP, blocking the heat flow in the process, which is translated as a decrease in the differential signal (**blue curve**), (**b**) a similar experiment using an analogue—histamine—demonstrates a different behavior. Histamine does not bind to the MIP, and a small increase rather than a decrease in the differential signal can be observed.

The single-shot setup was left to stabilize in air without any fluid being added to the central cavity. When two droplets of whole blood (80 µL) spiked up to a concentration of 1.0 µM of serotonin were added to the central cavity, an increase in the non-regulated temperatures for both the NIP and the MIP were observed. The temperature of the NIP side of the sensor increased from 33.91 ± 0.11 to 35.32 ± 0.04 °C, the temperature of the MIP side of the sensor increased from 34.01 ± 0.05 to 34.85 ± 0.06 °C (Figure 6a). The simultaneous increase in the temperature of both flow chambers can be accounted by the slightly better thermal conductivity of blood in comparison to air. However, due to the serotonin binding to the nanocavities of the MIP, the effect is less pronounced in comparison to the signal in the NIP channel which is translated in a steep decrease in the differential signal (blue curve). 

The selectivity test, exposing both channels to blood samples spiked with 1.0 µM of the analogue neurotransmitter histamine, is shown in Figure 6b. Addition of the whole blood samples leads to an increase in the non-regulated temperature in the NIP channel from 33.39 ± 0.05 to 34.64 ± 0.03 °C, which is comparable to the experiment with the target. The signal inside the MIP channel increases in a similar manner from 33.37 ± 0.04 to 34.79 ± 0.05 °C which can be explained by the fact that the additionally present histamine does not significantly bind to the MIP. 

**Figure 7:**
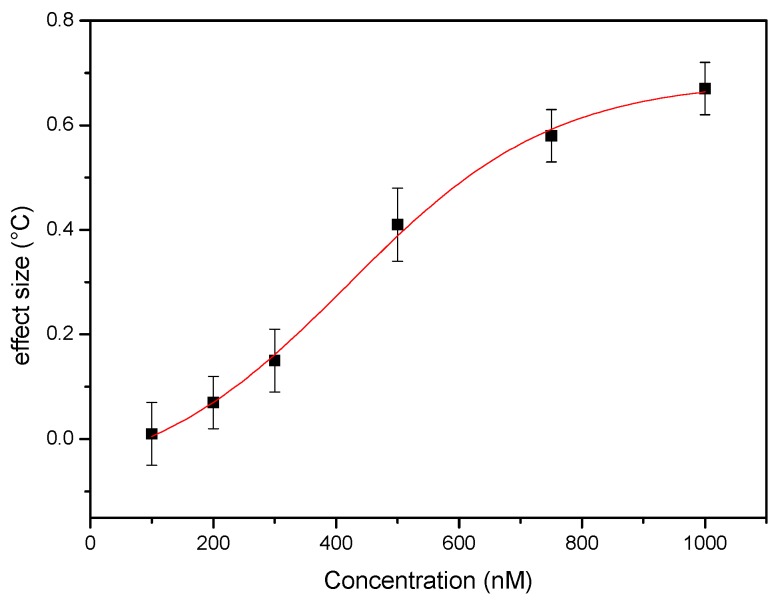
Dose-response curve obtained by analyzing the response of single shot devices to whole blood samples spike with increasing concentrations of serotonin. The absolute change in the differential signal is presented in function of the spiking concentration. The red curve represents an allometric dose-response curve (R^2^ = 0.996). These data indicate that it is possible to qualitatively detect fluctuations in the concentration of serotonin in whole blood samples.

In order to verify whether the single shot device is able to register fluctuations in serotonin concentration in a quantitative manner, the single shot experiment was repeated with blood samples spiked with increasing concentrations of serotonin, as described in Section 2.5. The results of these measurements are summarized in the dose-response curve shown in Figure 7, where the absolute differential effect size is presented in function of the spiking concentration. The data were analyzed using OriginPro and follow an allometric dose-response fit (R^2^ = 0.996), indicating that the single shot device is able to pick up clinically relevant differences in serotonin concentration in whole blood.

## 4. Conclusions

The data presented in this article demonstrate that the proposed sensor platform is able to detect clinically relevant fluctuations in the serotonin concentration of whole blood samples. The setup, based on combining synthetic MIP receptors with the novel heat-transfer method (HTM), was previously demonstrated in buffer solutions. However, the effect in biological samples remained to be evaluated. In this contribution, a first proof-of-principle experiment was performed with a four-chamber MIP-based sensor platform that could successfully detect serotonin qualitatively and quantitatively in spiked whole blood samples while being able to selectively distinguish between histamine and serotonin. The ultimate goal was to use these measurements for routine tests and therefore, a disposable single shot device was developed. The novel device featured a capillary pumping unit, making it fully integrated as the use of syringes was no longer required and the setup was miniaturized, requiring only 80 µL of sample, corresponding to two drops of blood, making the setup compatible with standard blood sampling techniques such as a blood lancet. With this design, the MIP and its reference NIP can be measured simultaneously in a “single-shot” measurement. Since there is no need for a stabilization step, the measurement time can be reduced to the order of minutes. The sensor platform was demonstrated to be both specific, as only the MIP reacted towards serotonin, and selective, proven by means of a selectivity experiment with its competitor neurotransmitter histamine. Additionally, the sensor was able to quantitatively measure fluctuations in the clinically relevant concentration regime. In summary, this work introduces the first hands-on device for the thermal detection of neurotransmitters with MIP-type receptors. The proposed sensor platform has great potential to be used in clinical research and diagnosis of e.g., early stage depression as recent evidence has shown that although serotonin cannot penetrate the blood-brain barrier and therefore has to be synthesized locally. Whole blood levels of serotonin can be used to be study and diagnose clinical depression. In order to truly use the technique for point-of-care sensing the readout equipment needs to be scaled down further, and improvements can be made to the synthetic receptor layer in order to improve the inter-sample variability as well as the sensitivity and dynamic range of the sensor.
